# Void Content Determination of Carbon Fiber Reinforced Polymers: A Comparison between Destructive and Non-Destructive Methods

**DOI:** 10.3390/polym14061212

**Published:** 2022-03-17

**Authors:** Moustafa Elkolali, Liebert Parreiras Nogueira, Per Ola Rønning, Alex Alcocer

**Affiliations:** 1 Department of Mechanical, Electronic and Chemical Engineering, Oslo Metropolitan University, 0130 Oslo, Norway; peror@oslomet.no (P.O.R.); alepen@oslomet.no (A.A.); 2 Department of Biomaterials, Institute of Clinical Dentistry, University of Oslo, 0317 Oslo, Norway; l.p.nogueira@odont.uio.no

**Keywords:** carbon fibers, porosity, void content, MicroCT analysis, chemical analysis, acid digestion

## Abstract

The properties of composite materials are highly dependent on the fiber and matrix fraction and on the porosity resulting from micro voids. This paper addresses void content characterization and the constituent content of composite materials by resorting to a comparison of destructive and non-destructive methods. The work presents the detailed procedures of two destructive methods, using acid digestion of epoxy resins matrices, and compares their processes. It also presents the results of a non-destructive method, by means of Micro Computed Tomography (MicroCT). The results of both destructive and non-destructive methods are compared, and a recommendation is made based on the application and the type of composite being analyzed. The MicroCT showed better and more consistent results in detecting voids in the material, while the acid digestion tests provided better results about the fiber and matrix percentage. Exported results from the MicroCT scanning with actual locations of voids were used in numerical analysis, to examine the feasibility of using them, whether by developing models that map damage in the proximity of the void, or by developing models that predict the properties of the entire material with respect to the content, shape, and distribution in the material.

## 1. Introduction

Carbon fiber composites are being used in a steadily increasing number of applications, including piping, pressure housings for underwater robotic vehicles, and other marine structural components [1]. Carbon fiber composites exhibit excellent mechanical properties, and in many cases allow to reduce the weight of parts in a substantial manner. In order to ensure a good and reliable performance of the Carbon Fiber Reinforced Polymer (CFRP) structure, it is crucial to ensure a low level of void content. Voids are formed during the coating of fibers with resin as a result of the inability of the resin to fully replace air from the fiber surface. Another common cause of void content in the laminate is the entrapment of air bubbles and volatiles during lamination, particularly when using the filament winding manufacturing method. Many other factors affect the formation of voids and the quality of the composite, such as the viscosity of the resin, the temperature of the resin during manufacturing, and processing parameters such as curing cycles, pressure, and time [2,3,4].

Voids are mainly spherical, which is due to the gelation phase that the matrix goes through during polymerization at the curing process [5]. Several methods can be used to reduce the void content during manufacturing and help reduce their total volume and size in the material, such as autoclave curing and vacuum bagging. However, voids cannot be eliminated entirely [6].

**Figure 1:**
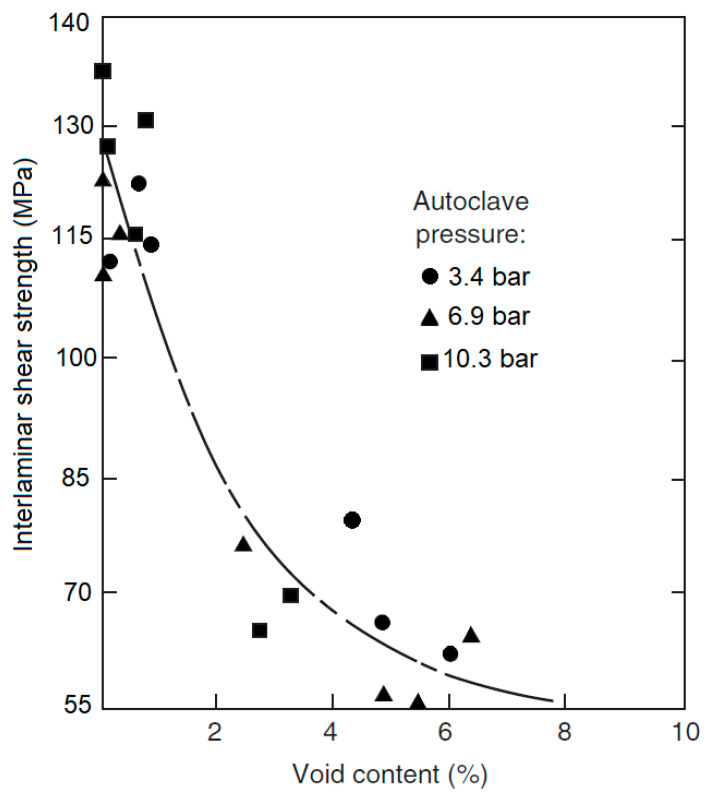
Effect of void content on the interlaminar shear strength, according to [9].

Voids act as stress intensifiers and have a huge impact on the mechanical properties of the material. Stress concentration increase by a factor of up to 1.85 near the edges of the voids [7]. A high void content laminate will have an easier crack propagation as well as a weaker bonding between the fiber and the matrix [6]. This results in a reduction in the mechanical properties of the material and may result in catastrophic and premature failure. According to [8], the inter-laminar shear maximum strength is lowered by 7% for only 1% of void content in the composite material. Other material properties such as in-plane shear, tensile and flexural strengths, and moduli are also greatly affected by the void content [3]. Figure 1 shows a relation between the void content percentage and the inter-laminar shear stress for a 12-ply unidirectional laminate, that has between 60 and 70% fiber content [9].

Not only do voids have a significant effect on the mechanical properties of the material, but they also facilitate abrasion between the fibers resulting in fiber damage [10], as well as increase the vulnerability to water penetration and weathering effects [11], which is crucial in marine applications. For these reasons, the knowledge of void content is important for the quality control of a manufacturing technique and the prediction of the properties of the manufactured component. A relation was noted by [12], by using means of ultrasonic testing, that approximately up to 1.5% of the void content is mainly volatile-induced voids, and the remaining of the void content, if there is any, is caused by the entrapment of air bubbles between the laminae.

Another main factor that affects any composite properties is the fiber and matrix content. They have the dominant effect on the mechanical properties of the composite material. Although the Rule of Mixture is not entirely accurate, it provides an estimation of the elastic properties of the composite material [9]. Hence, the fiber and matrix content are two essential qualities to measure. Approaches to measure the fiber-to-matrix ratio are standardized in both automotive and aerospace industries [13]. Gravimetric tests are performed on samples of the manufactured material to eliminate the matrix content and measure the mass of the sample before and after the removal of the matrix. If the densities of the fibers and matrix used, as well as the volume of the sample before the removal of the matrix are known, the volume of any void content present can be also calculated. Therefore, the physical properties of the fibers and the matrix has to be known accurately, whether to calculate their ratio or to calculate the void content in the composite [14]. Non-physical negative values of void content have been reported by [15] due to wrong density inputs.

Recent progress in numerical techniques and computational power have enabled more involvement of numerical modelling in the study of voids in composites [14]. Many numerical methods have been used lately to study the effects of void content on the material, but the experimental approach had the biggest share of contributions to this field [14]. However, in many studies, it was observed that not only the void content determines their effect on the material, but also their morphology (shape), location, size, and distribution contribute to the impact on the material mechanical properties [16,17,18,19]. Using non-destructive testing that can provide full 3D analysis, such as MicroCT scanning, and Finite Element Modelling (FEM), facilitated achieving such analyses and conclusions. Ref. [20] introduced an approach that considers the voids volume fraction by importing the actual geometry from MicroCT scanning. A reduction in the matrix properties was done based on a void compensating factor $\frac{V_{m}}{V_{m}+V_{v}}$, where $V_{m}$ and $V_{v}$ are the volumes of the matrix and voids, respectively. The results of the finite model, tensile modulus, and in-plane shear modulus, although performed on a micro-scale, have matched the experimental results [14,20]. Others have used a different approach for the prediction of strength for composites with voids, such as in Ref. [21]. They developed a Finite Element (FE) 3D model based on the failure criterion of Ref. [22]. They concluded that the criteria of judging the quality of the manufactured composite, based on the volumetric percentage solely is not enough. Despite that, in some of the tested samples, the porosity was only 0.1% and the strength reduction was more than 50% because some of the pores were in critical areas. 

The aim of this study is to perform two types of standard destructive tests and one type of standard non-destructive testing, on samples cut from filament wound tube, in order to know the fiber, matrix, as well as volume percent of any voids present and compare the results of the tests. An evaluation of the two methods of destructive tests is also an aim of the work to make a recommendation on which method has higher accuracy. An evaluation of the non-destructive test method used is also carried out, by assessing the resolution of the results and the ability to perform and export 3D analysis. Scanned and reconstructed data of the material tested was exported to be analyzed using numerical analysis, allowing further research to be done on the prediction of the laminate strengths using numerical modelling and the comparison of these predictions to mechanical testing. Another aim of the study is to compare the results of the destructive and non-destructive tests, in terms of accuracy, simplicity, and availability.

The results of this work will be also used in the future within the framework of OASYS research project to design and manufacture the hull of a deep autonomous underwater vehicle, using a comparable stacking sequence as the one tested, by adapting the mechanical properties of the composite, such as the longitudinal and transverse modulus and strength according to the fiber, matrix, and voids content.

## 2. Methodology

### 2.1. Destructive Testing

There are various methods for inspecting the quality of the composite manufactured part that includes both non-destructive and destructive tests. The destructive methods in general are more suitable for quality control processes, since it allows the testing of large samples, and it is relatively fast and more economical compared to the non-destructive testing techniques [14]. One example of destructive methods is matrix ignition, also known as resin burn-off [13]. In the burn-off method, the matrix is physically removed entirely from the composite test sample by using a Muffle Furnace or a Nitrogen-Purging Furnace to increase the temperature of the sample to 500–600 °C, consecutive to measuring its weight and volume. Nitrogen is used to prevent the oxidation of carbon fibers [13]. The sample is then cooled and measured again for its volume and weight, to know the volumetric percentage of the matrix and voids. This method is used generally with polymer-matrix composites (PMC) fibers that do not lose weight at high temperatures, such as ceramics and glass [23]. This method has some drawbacks, e.g., it cannot be used with carbon fibers unless the temperature is closely controlled (±5%) to prevent the char of the fibers [23], as well as not being as simple as other destructive tests such as matrix digestion, since it requires some expensive apparatus, for instance, a Nitrogen-Purging Furnace.

Matrix digestion, commonly known as acid digestion, was favored in this work as a destructive method to determine the constituent content of the composite. Several acids are recommended by Ref. [23] depending on the type of the matrix and reinforcement in the material. For non-metal matrices and carbon fiber reinforcements, there are three standard procedures using three different digestion solvents: one involves the use of Sulfuric Acid, another Nitric Acid, and the third uses a solution of Potassium Hydroxide and Ethylene Glycol. Two test procedures were carried out in this work using Nitric Acid and Sulfuric Acid. The tests were done according to the recommendations of Ref. [23]. The error of the void content resulting from destructive testing of the composite is no less than ±0.5%, even for the best results [24].

#### 2.1.1. Procedures

##### Specimen Preparation

All the samples were cut using diamond-tipped tooling to prevent any damage to the material, such as delamination, and to avoid trapping air in the samples during the cutting process. Six non-identical samples were tested in each method, all cut from the same specimen. The minimum samples required to be tested is three samples in each method, according to ASTM recommendation [23].

The samples used in the analyses were all cut from a filament wound tube that has ply angles of [12_3_/85_2_]_s_. The composite used in this work consists of Toray^®^ (Tokyo, Japan) T-700 Standard Modulus Carbon Fibers, and the matrix used is epoxy Hexion^®^ (Columbus, OH, USA) EPIKOTE 828 as a resin mixed with EPIKURE 866 curing agent and EPIKURE 101 catalyst with the ratio of 100:80:1.5 by weight, respectively. The density of the used fibers is 1.760 kg/m^3^ and the density of the matrix is 1.1804 kg/m^3^. The filament wound tube was cured at 90 °C for 1.5 h, then at 150 °C for 2.5 h. A thermo-shrinkable folio tape, designed to be used with epoxy resins, was then wrapped in direct contact with the external lamina and compressed the tube over the mandrel when it was put in the oven for curing.

Before the testing procedures, the density of the samples was measured. The samples were dried in an airflow oven and cooled in a desiccator to prevent any false mass readings due to moisture content. The mass of each sample was then measured to the nearest 0.1 mg using an analytical balance, “Mettler Toledo-New Classic MF”. The volumes of the samples however were measured using a more complicated approach, by using the buoyancy principle. The samples were suspended using a thin copper wire that has a diameter of 0.15 mm and submerged in temperature monitored distilled water, and the change of weight was recorded before and after the submerging, according to Ref. [25]. The distilled water used had a temperature of 25.1 °C. The volume of the sample was then calculated using Equation (1), where $V_{s}$ is the volume of the sample, $V_{SW}$ is the volume of the submerged suspending thin wire, $M_{i}$ is the mass of the sample, $M_{S}$ is the submerged mass of the sample, $\rho_{DW}$ is the density of the fluid used to provide the buoyant case, which is distilled water at 25.1 °C in this experiment and equal to 0.99708 kg/m^3^, according to Ref. [25].

(1) $$V_{S}=\left[\left(M_{i}-M_{s}\right)/\rho_{DW}\right]-V_{SW}$$

Table 1 shows the properties of each tested sample. After the mass and volume of each sample were measured, samples were dried and then stored in a 25 °C environment with 50% relative humidity, for 24 h prior to the tests.

##### Acid Digestion Tests Using Sulfuric Acid

**Figure 2:**
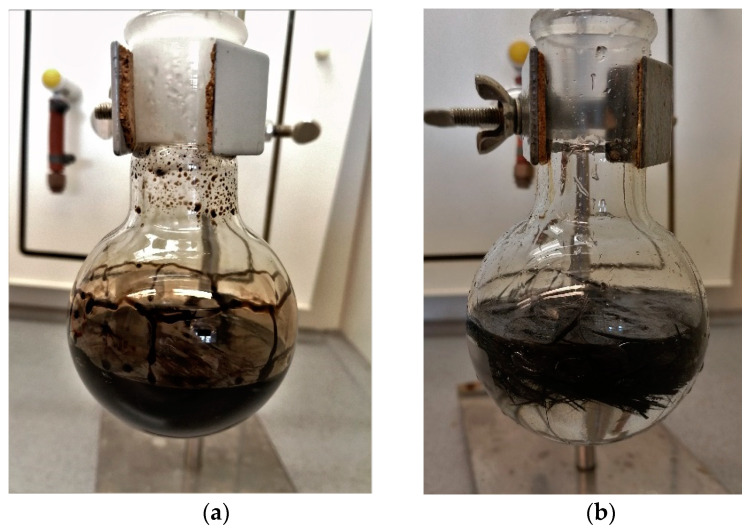
(**a**) Carbon/Epoxy sample during sulfuric acid digestion; (**b**) after the addition of Hydrogen Peroxide Description of what is contained in the second panel.

The other six samples were put in separate beakers, and 30 mL of concentrated sulfuric acid (98%) was added into each beaker. The mixture was heated using electric heaters until fumes came out of the beaker, the color of the acid turned dark black, indicating that the reaction was accelerated by the applied heat and that the sulfuric acid has etched the matrix around the fibers, as shown in Figure 2a. The beakers were left for approximately 15 min until no further change in color was noticed, as shown in Figure 2b.

**Figure 3:**
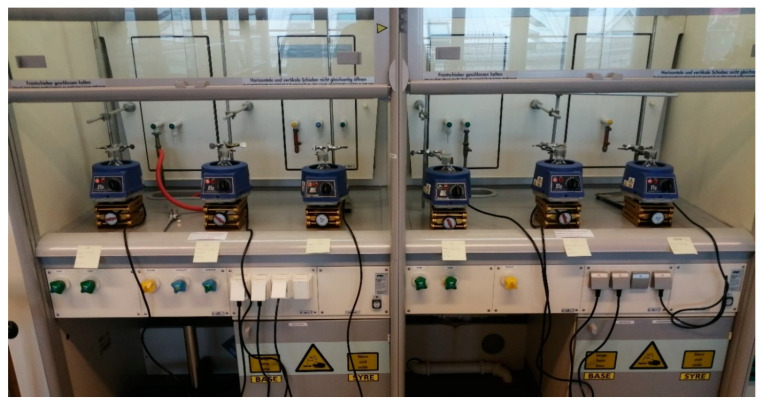
Six samples being simultaneously tested in the vacuum fume hood.

Then, 40–50 mL of 30% hydrogen peroxide was added into the beaker, producing what is known as the “Piranha Solution” a very strong oxidizing agent which will oxidize the epoxy matrix and make it hydrophilic. The dark color resulting from the etching process will dramatically fade and the remaining fibers were visible floating on top of the solution, and it was clear that there was no undigested material in the beakers, as shown in Figure 2b. The reaction with the hydrogen peroxide is highly exothermic and results in acid fumes and splashing high-temperature acid droplets. Hence, the experiments were performed in a fume hood, as shown in Figure 3. 

**Figure 4:**
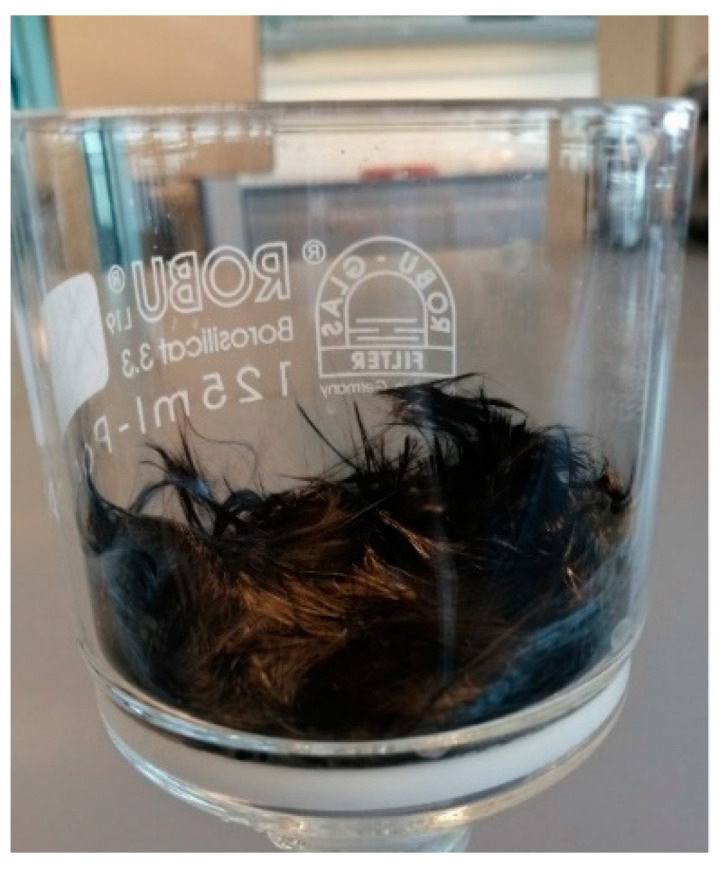
Remaining carbon fibers in the filter after the etching process.

The beakers were cooled in an ice bath for safe handling and were then vacuum filtered in a pre-weighted, pre-rinsed with diluted hydrochloric acid and distilled water, and oven-dried sintered glass filter, as shown in Figure 4. Glass filters offer high chemical resistance and high-temperature resistance in comparison with normal paper filters, which cannot be used in this application. The used filters have a porosity size of 10–16 µm. After filtration, the samples were then washed carefully in the filters using distilled water and acetone and placed in a drying oven at a temperature of 120 °C for 12 h. Finally, the filters containing the samples were weighed to the nearest 0.1 mg.

##### Acid Digestion Tests Using Nitric Acid

**Figure 5:**
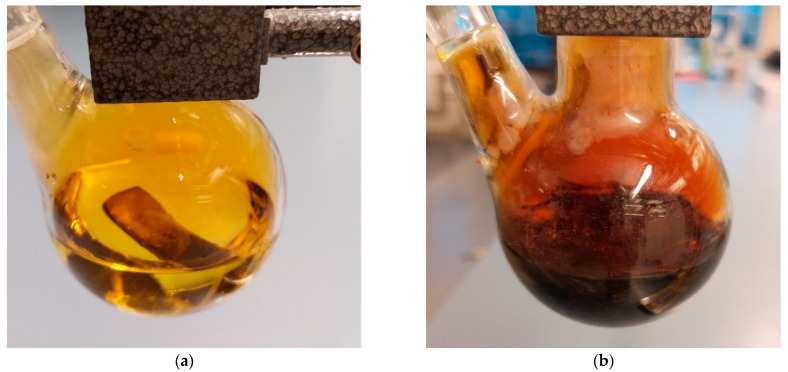
Sample in nitric acid; (**a**) after 6 h of digestion; (**b**) after 18 h of digestion.

Six samples were put in separate round-bottom flasks fitted with reflux condensers, each flask containing 30 mL of 65% nitric acid. The flasks were then put in a hot water bath at 60 °C to ensure constant and controlled heating. The samples were left for 6 h first, but digestion was ineffective, as shown in Figure 5a. The temperature of the hot bath was then increased to 80 °C, another 30 mL of nitric acid was added to each flask, and the samples were left for another 12 h. Etching of the material was more complete, but traces of non-digested material still existed, as shown in Figure 5b.

Unfortunately, these traces are very hard to distinguish in the flasks, until the material is removed. Any non-digested material was put back into the acid to continue the digestion process. The non-digested material should be visible as residues of the composite, as shown in Figure 5. The total time of the process was around 40 h. The fibers were vacuum filtered in pre-weighted sintered glass filters, similar to the procedure using sulfuric acid. The fibers were washed in the filter crucibles with distilled water and acetone several times before the filters were placed in a drying oven at a temperature of 120 °C for approximately 12 h. Finally, the filters with the filtered fibers were weighed to the nearest 0.1 mg, using an analytical scale.

##### Calculations

After performing the experiments, the fiber and matrix content, volume and weight, can be calculated according to Equations (2)–(6), while void content can be calculated according to Equation (7) [11,23].

(2) $$M_{f}=M_{cf}-M_{c},$$

(3) $$W_{f}=\frac{M_{f}}{M_{i}}\times 100,$$

(4) $$V_{f}=\frac{M_{f}}{M_{i}}\times \frac{\rho_{i}}{\rho_{f}}\times 100,$$

(5) $$W_{m}=\frac{M_{i}-M_{f}}{M_{i}}\times 100,$$

(6) $$V_{m}=\frac{M_{i}-M_{f}}{M_{i}}\times \frac{\rho_{i}}{\rho_{m}}\times 100,$$

(7) $$V_{v}=100-\left(V_{f}+V_{m}\right),$$

where $W_{f}$ and $W_{m}$ are the percentages of the weight of the fibers and the matrix in the composite accordingly. $V_{f}$, $V_{m}$, and $V_{v}$ are the volumetric percentages of the fibers, matrix, and voids in the composite accordingly. $M_{f}$, $M_{c}$, $M_{cf}$, and $M_{i}$ are the mass of the sample after digestion, the mass of the dry filter, the mass of the filter with the fibers remaining from the chemical reactions, and the mass of the sample before digestion. $\rho_{f}$, $\rho_{m}$ and $\rho_{i}$ are the densities of the fibers, matrix, and the sample, respectively.

#### 2.1.2. Results of Destructive Testing

The results of the tested samples should be very similar; hence six samples were tested in each analysis to detect any error that may be present in any of the samples. Errors may show up during the preparation of the samples, such as delamination due to the cutting process, giving false readings about the presence of voids. They may appear also during the experiment such as the presence of non-digested matrix or any change in fiber weight before and after the tests.

Ideally, the mass of the fibers should be identical before and after testing, and the mass of the matrix should be zero after testing. However, due to imperfect testing conditions or procedures, these two properties may change. In order to compensate for these two factors, the full procedures of experiments can be repeated on two blanks. One blank consists entirely of fibers, and the other blank consists entirely of the cured matrix. The change in the mass in the fibers and the matrix, if it is reproducible, can be taken as a correction factor to be added or subtracted from all the actual samples. Only changes bigger than 0.5% should be considered [23].

**Figure 6:**
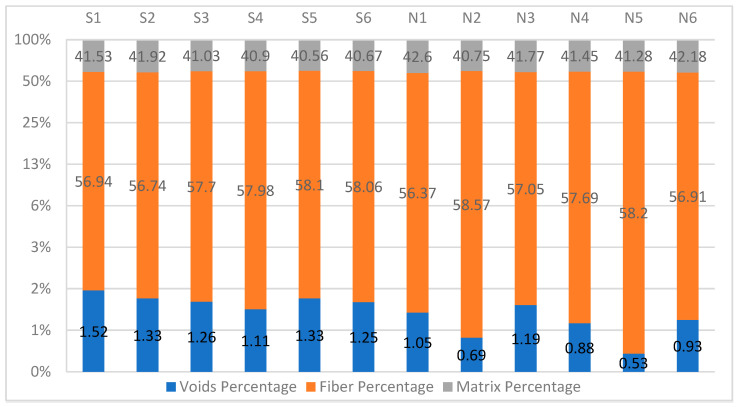
Volume constituent content by acid digestion with a logarithmic scale.

**Figure 7:**
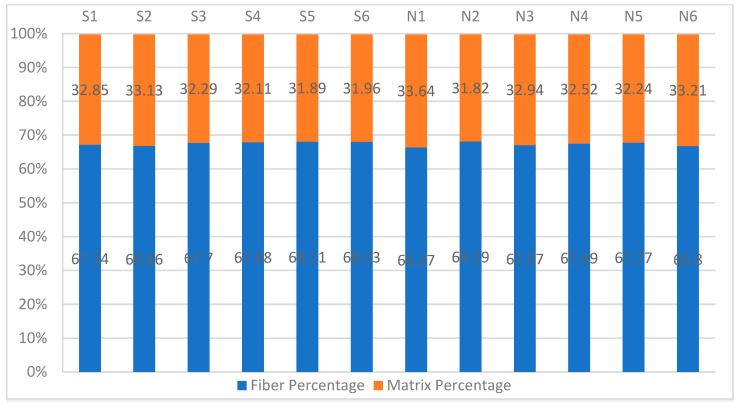
Mass constituent content by acid digestion.

The results of each of the 12 tested samples from the acid digestions tests were similar. Figure 6 and Figure 7 show the percentage of the constituent content of the material tested using acid digestion by mass and volume on a logarithmic scale, without taking the fiber or matrix correction factors into count. However, the void percentage of the samples tested using the nitric acid approach had a bigger variation than the ones tested using the sulfuric acid approach. This supports the observation that sulfuric acid is a more efficient and faster digestion agent for the polymers studied in this experiment. None of the results exceeded the 2% limit recommended by Ref. [9].

Since this is a gravimetric analysis, the average values, Standard Deviation (SD), and Coefficient of Variation (CoV) were calculated and shown in Table 2. Both the CoV and the SD are low in both approaches volume contents of the samples, which is a good indication that none of the samples had a flawed test result, like a partially digested sample, as explained earlier.

### 2.2. Non-Destructive Testing

#### 2.2.1. Approach and Methodology

In most of the above tests, the work described used void content figures obtained by destructive means. These are essentially rather laborious point-by-point measurements giving an average value over a finite volume of material whilst they cannot give some guidance as to the variation of voids across the material. Furthermore, being destructive, they cannot be accompanied by mechanical testing on the same test samples. Moreover, they do not take into count all the voids and the micro voids in the material [26], and they do not provide information about the size or the shape of the voids.

It is clear therefore that a more detailed investigation of the effect of voids on mechanical performance requires accurate non-destructive tests. Different non-destructive methods have been used for measuring the void characteristics, such as Ultrasonic and MicroCT testing. Ultrasonic testing uses controlled ultrasonic energy. Attenuation or dispersion of the pulse will occur if there is any discontinuity in the material [14]. Ultrasonic testing is precise and possible for inspection during service [14]. However, it requires a coupling agent such as oil or water, although late research has been done using noncontact air-coupled ultrasonic testing [27,28]. The biggest drawback of ultrasonic testing is that it provides poor feedback about the morphology of the voids [14]. The MicroCT scanning, however, uses X-ray to generate 3D images of the samples. Briefly, the sample is scanned around its central axis, while 2D images, also called projections, are taken in small steps. The resulting dataset will be then reconstructed into virtual 2D slices, which can be visualized in any orientation, as well as 3D rendered, and have its structures quantified [29]. It is able to provide a full 3D analysis of the morphology, distribution, and location of the voids. MicroCT is not without any disadvantages, though. The resolution of the scanning is dependent mostly on the size of the sample. The smaller the scanned specimen, the higher the resolution that can be achieved. Although large devices can be found, they are not very common [14].

A MicroCT scanner was used for this analysis in order to capture voids down to 3.5 µm in diameter. The specimens were scanned using a Skyscan 1172 MicroCT [30]. Samples to be scanned were cut to the dimensions of approximately 6.0 mm × 4.0 mm using a diamond-tipped tooling, same as the acid digestion samples, to prevent damage to them. The parameters used for scanning were: 49 kV and 190 µA; exposure time of 240 ms per projection, and frame averaging of 5 images per projection, making out a total acquisition time per projection of 1200 ms. A total of 880 projections were evenly acquired by a charge-coupled device (CCD) camera while the sample was rotated around 360 degrees, at a field of view of 2000 × 1332 pixels. The projections taken from the scanner were then reconstructed using the reconstruction software NRecon [31], with a final pixel size of 3.5 µm. Further segmentation and analysis were performed using CTAn software [32]. Finally, three-dimensional rendering of the images was performed with “Dragonfly” software [33].

After the acquisition and reconstruction of the datasets, quantification of pores could be performed. For this, the images were segmented using the procedure described by [34], so that pores could be separated from the fibers, matrix, and background. The sample volume, number of closed pores, volume of closed pores, and closed porosity were analyzed in this work. Closed porosity is defined as the ratio between the pores that do not have any connection with the boundaries of the sample and the analyzed sample volume. Pore size distribution was also quantified. The pore size is calculated based on the local thickness, where local thickness is evaluated as the diameter of a hypothetical sphere that fits within each surface point of the pore [35].

The specimens used in the non-destructive testing was prepared in the same procedures as the destructive tests’ specimens, as explained in Section Specimen Preparation, except that the specimens were cut in smaller size to get higher resolution from the scan, as mentioned later. They were cut from the same filament wound tube as the destructive testing samples. This allows the comparison of the two results.

#### 2.2.2. Results of Non-Destructive Testing

**Figure 8:**
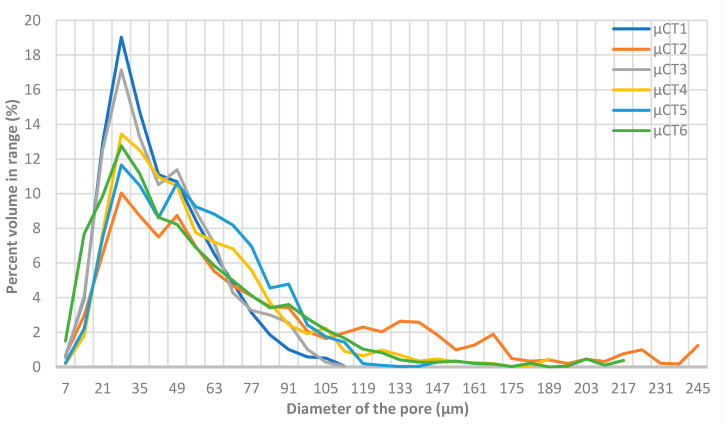
Graph representing pore size distribution in the six scanned samples.

The MicroCT scanned samples showed a much lower standard deviation as shown in Table 3. The average porosity was 1.53% with a standard deviation of 0.04%”. The majority of the pores have sizes that lie within the range of 24.5–31.5 µm in diameter, as shown in Figure 8, which is a low range.

**Figure 9:**
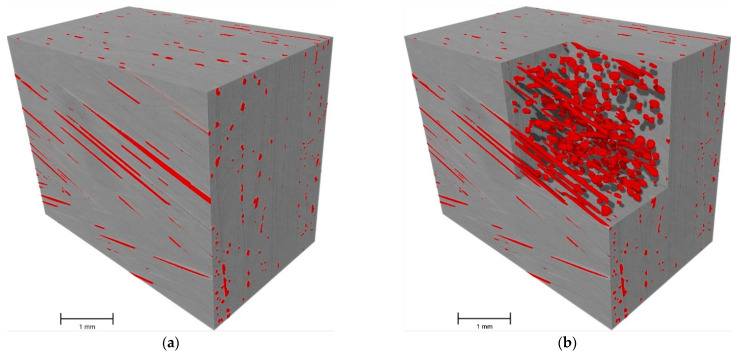
Scanned sample “µCT 2” showing the composite in grey and the voids in red; (**a**) the scanned sample, (**b**) a section cut showing the voids in the sample.

The MicroCT scan results are close to the acid digestion ones. Unfortunately, the scan is not capable of distinguishing the difference between the two material components clearly enough, due to their very similar density. Given that the difference between the densities of the fiber and the matrix is not a considerable amount, it was not possible to obtain the mass constituent content of the fibers and matrix in the scanned samples, using the MicroCT scan, which is one of the main drawbacks of using MicroCT scanning in such analysis. In some cases, a contrast agent is added to the resin before manufacturing to increase its absorption coefficient, which will make it more distinguishable while scanning the samples [36]. On the other hand, the MicroCT scans provided a resolution down to 3.5 µm/pixel allowing the detection of all the pores bigger than approximately 10 µm, as well as their size and their distribution. Figure 9 shows a reconstructed, rendered image of one of the scanned samples. Not only did it provide good resolution, but also showed higher precision, as shown in Table 3. More importantly, it allowed further inspection of the pores using finite element analysis, as performed in the following section.

#### 2.2.3. Numerical Modelling

The numerical modeling is performed on one isolated layer of one of the MicroCT scanned samples. MicroCT provided a reliable geometry input of the morphology and distribution of the voids. This model focuses more on the voids and their short-range effects. This does not include long-range effect on properties, such as effect on the fracture toughness. The numerical model, in this case, is done to show how much the voids will contribute to the failure of the material under longitudinal and transverse loading, and, if failure is initiated, whether it will occur at or in the proximity of the voids. In other words, by creating a damage map around the voids, Ref. [37] found a direct correlation between fiber failure and the existing voids in the material. Fiber breaks were found to occur up to five times more immediately next to existing voids. 

**Figure 10:**
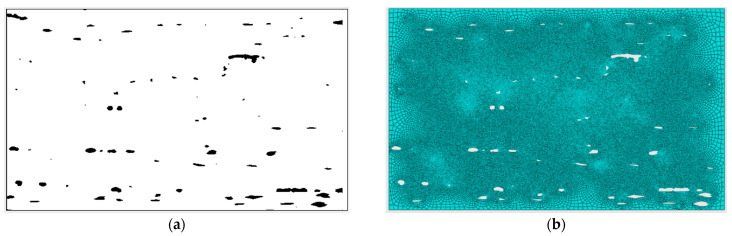
Model used in ABAQUS software; (**a**) Exported binary image from the MicroCT scan, (**b**) The meshed created model.

The layer that has the voids was exported from the scan and imported into ABAQUS software [38]. The damage model used is Hashin’s Model [39]. The damage model enabled calculating the parameters of the homogenized damage. Ref. [40] did similar research on predicting the tensile and compressive strengths of a uni-directional laminate with void content ranging from 0.33 to 1.5% using FE modelling. Hashin’s theory considered the damage initiation in a given fiber-reinforced composite, by one or more of four criteria. These criteria are fiber tension, fiber compression, matrix tension, and matrix compression [39]. Equations (8)–(11) represent the failure in the composite material using the four criteria mentioned earlier. Table 4 shows the values used for the numerical model obtained from the datasheet of the material used in manufacturing and their denotations. $\sigma_{11}$, $\sigma_{22}$, and $\tau_{12}$ are the normal and shear components of the effective stress matrix. Damage is initiated if any of the Hashin factors is bigger than 1. The model proposed by Hashin recommends setting α to 1 [39]. A damage evolution model has also been defined so that the maximum variable of these factors does not exceed 1.0. Figure 10 shows the binary image exported from the scan and the meshed model created in ABAQUS.

The model is deformable shell and the element type used in the analyses is a general purpose Stress/Strain shell element with four nodes (S4R). The part is fixed from one edge and loaded from the opposing edge, both in the longitudinal and transverse direction of the composite, in order to inspect the failure locations for both the matrix and fibers. The loads applied are purely tensile loads, where no other in-plane loads were applied.

(8) $$F_{f}^{t}={\left(\frac{\sigma_{11}}{X^{T}}\right)}^{2}+\alpha {\left(\frac{\tau_{12}}{S^{L}}\right)}^{2},$$

(9) $$F_{f}^{c}={\left(\frac{\sigma_{11}}{X^{C}}\right)}^{2},$$

(10) $$F_{m}^{t}={\left(\frac{\sigma_{22}}{Y^{T}}\right)}^{2}+\alpha {\left(\frac{\tau_{12}}{S^{L}}\right)}^{2},$$

(11) $$F_{m}^{c}={\left(\frac{\sigma_{22}}{2S^{T}}\right)}^{2}+\frac{\sigma_{22}}{Y^{c}}\left[{\left(\frac{Y^{C}}{S^{L}}\right)}^{2}-1\right]+{\left(\frac{\tau_{12}}{S^{L}}\right)}^{2}.$$

**Figure 11:**
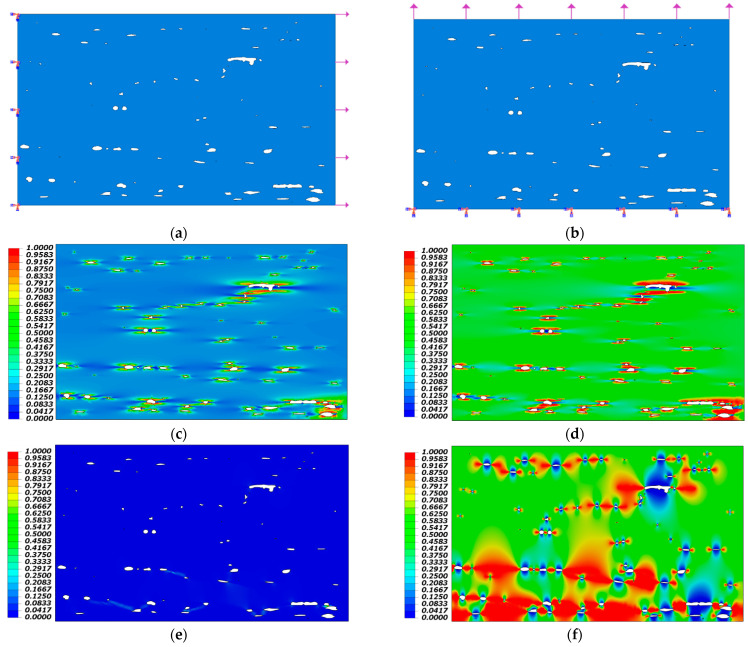
Results of numerical simulation showing: (**a**) load direction in longitudinal direction; (**b**) load direction in the transverse direction; (**c**) fiber failure under longitudinal loading; (**d**) matrix failure under longitudinal loading; (**e**) fiber failure under transverse loading; (**f**) matrix failure under transverse loading.

Two simulations were performed: one simulating pure longitudinal tensile load, and the other simulating transverse tensile load. The results showed that, although the loading is safe to be applied on the material based on its property, both matrix and fiber failures initiate at the voids whether in the longitudinal or the transverse loading. Figure 11a–f shows the results of the two simulations. Damage can be seen occurring in the adjoining regions of voids, although that there is no damage in the rest of the regions. This confirms the results of Ref. [37], who found that voids contribute greatly not only to matrix cracks but also to fiber breakage in filament wound composites.

## 3. Discussion and Conclusions

Manufactured defects such as voids can have a great impact on the properties of the composites. Even though it is not possible to eliminate them entirely, it is possible to include their realistic presence in the design, avoiding using both extremes of “worst case”, and “far optimistic” scenarios.

In this study, destructive and non-destructive testing was performed on CFRE composite, to evaluate the volumetric percentage of fibers, matrix, and voids. The samples were taken from a filament wound tube. The results will be incorporated in a design of a tube with a similar stacking sequence that will be used as a hull for an autonomous underwater vehicle.

Destructive testing was performed using acid digestion. Two approaches were used in acid digestion, one using sulfuric acid and the other by using nitric acid. The results of the mass content from the two approaches were similar. The average fiber/matrix mass ratio of the six samples of each approach were different only by 0.35%. The approach of using sulfuric acid proved to be much easier to be used. The time of the experiment did not exceed one hour, and it does not require the same lab equipment as the approach using nitric acid, while the material digestion using nitric acid took approximately 40 h. The results of the nitric acid approach showed a factor of variation that is almost three times the one of the sulfuric acid. This means that using sulfuric acid showed higher precision. However, it involves more safety hazards and requires more safety equipment. 

Acid digestion, specially using sulfuric acid, was simple, cheap, and better at identifying the mass constituent of the reinforcement and matrix, but the results were not as accurate as of the MicroCT scan in identifying the micro voids in the material and vice versa.

Accurate non-destructive testing was done by means of MicroCT scanning. In order to get to very low accuracy, 3.5 µm/pixel, the samples had to be cut into a very small size, approximately 70 mm^3^ each. This might not be feasible if a proper cutting CNC machine and proper tooling are not available. The MicroCT scanning however, was excellent in detecting any voids that have sizes bigger than ~3 µm, but unfortunately, it was not able to distinguish the difference between the matrix and the reinforcement clearly enough, due to the fact that the difference between them was not considerable enough. 

Another advantage of the MicroCT scan is that it enables the possibility of further analyses to be done on the sample. Numerical analysis was performed on an isolated layer of one of the samples, and the numerical testing clearly showed failure loci in the matrix and the reinforcement occurring at the voids in both longitudinal and transverse loading. A very critical factor that judges the accuracy of the results of the model is the choice of the correct damage model and the correct damage controlling parameters in the micro-scale, which should be measured based on the results of tests performed on a micro-scale. In future work, we intend to validate the FEM by mechanical testing of samples, and study the possibility of correlating the results from MicroCT scans to the prediction of the in-plane and out-of-plan strengths of the composite laminate.

## Figures and Tables

**Table 1:** Mass and volume of the 12 destructive tested samples.

Approach A1 (Sulfuric Acid)			Approach A2 (Nitric Acid)		
Sample Number	Sample Mass	Sample Volume	Sample Number	Sample Mass	Sample Volume
	mg	mm^3^		mg	mm^3^
S1	3239.7	2170.686	N1	2827.0	1891.270
S2	2226.3	1490.700	N2	2775.8	1836.209
S3	2755.0	1836.711	N3	2518.5	1682.254
S4	2554.4	1699.109	N4	2474.5	1644.850
S5	2846.4	1895.783	N5	2564.3	1696.501
S6	2355.8	1568.333	N6	2179.9	1453.987

**Table 2:** Statistics of sample tested by acid digestion.

		Volume Content		
		Average (%)	SD (%)	CoV
Sulfuric	Fiber	57.60%	0.602	1.044
	Matrix	41.11	0.526	1.280
	Void	1.30	0.134	10.295
Nitric	Fiber	57.46	0.836	1.455
	Matrix	41.66	0.660	1.582
	Void	0.87	0.238	27.231

**Table 3:** Results of samples tested with MicroCT scan.

Sample	Sample Volume (mm^3^)	Number of Closed Pores	Volume of Closed Pores (mm^3^)	Closed Porosity Percentage (%)
µCT 1	60.054	3040	0.930	1.550
µCT 2	60.054	3413	0.904	1.506
µCT 3	60.054	2965	0.914	1.522
µCT 4	60.054	1997	0.923	1.537
µCT 5	60.054	1818	0.889	1.480
µCT 6	60.054	6457	0.955	1.591
		Average (%)		1.531
		Standard deviation (%)		0.038
		Coefficient of variation (%)		2.478

**Table 4:** Properties of the material used in the numerical model.

Property		Explanation	Value	Unit
**Elastic properties**	$E_{11}$	Longitudinal modulus of elasticity.	135	GPa
	$E_{22}$	Transverse modulus of elasticity.	10	GPa
	$v_{12}$	Poisson’s ratio.	0.3	GPa
	$G_{12}$	In-plane shear modulus.	5.0	GPa
	$G_{13}$	Out-of-plan shear modulus.	5.0	GPa
	$G_{23}$		2.5	GPa
**Damage properties**	$X^{T}$	Ultimate longitudinal tensile strength.	1760	MPa
	$X^{C}$	Ultimate longitudinal compressive strength.	1570	MPa
	$Y^{T}$	Ultimate transverse tensile strength.	80	MPa
	$Y^{C}$	Ultimate transverse compressive strength.	250	MPa
	$S^{L}$	Ultimate longitudinal shear strength.	98	MPa
	$S^{T}$	Ultimate transverse shear strength.	50	MPa
	α	Factor ranging from 0 to 1 representing the input of the shear strength to the fiber tensile damage.	1	-
**Hashin factors**	$F_{f}^{t}$	Hashin fiber factor in tension.		-
	$F_{m}^{t}$	Hashin matrix factor in tension.		-
	$F_{f}^{c}$	Hashin fiber factor in compression.		-
	$F_{m}^{c}$	Hashin matrix factor in compression.		-

## Data Availability

Not applicable.
